# *Shewanella algae* Relatives Capable of Generating Electricity from Acetate Contribute to Coastal-Sediment Microbial Fuel Cells Treating Complex Organic Matter

**DOI:** 10.1264/jsme2.ME19161

**Published:** 2020-03-06

**Authors:** Yoshino Inohana, Shohei Katsuya, Ryota Koga, Atsushi Kouzuma, Kazuya Watanabe

**Affiliations:** 1 School of Life Sciences, Tokyo University of Pharmacy and Life Sciences, 1432–1 Horinouchi, Hachioji 192–0392, Tokyo, Japan

**Keywords:** coastal sediment, microbial fuel cell, metabarcoding, exoelectrogen, electrode-plate culture

## Abstract

To identify exoelectrogens involved in the generation of electricity from complex organic matter in coastal sediment (CS) microbial fuel cells (MFCs), MFCs were inoculated with CS obtained from tidal flats and estuaries in the Tokyo bay and supplemented with starch, peptone, and fish extract as substrates. Power output was dependent on the CS used as inocula and ranged between 100 and 600 mW m^–2^ (based on the projected area of the anode). Analyses of anode microbiomes using 16S rRNA gene amplicons revealed that the read abundance of some bacteria, including those related to *Shewanella algae*, positively correlated with power outputs from MFCs. Some fermentative bacteria were also detected as major populations in anode microbiomes. A bacterial strain related to *S. algae* was isolated from MFC using an electrode plate-culture device, and pure-culture experiments demonstrated that this strain exhibited the ability to generate electricity from organic acids, including acetate. These results suggest that acetate-oxidizing *S. algae* relatives generate electricity from fermentation products in CS-MFCs that decompose complex organic matter.

A microbial fuel cell (MFC) is a type of bioelectrochemical system (BES) that exploits living microbes for the conversion of organic matter into electricity (Logan *et al.*, 2006). Bioreactor-type MFCs have been developed for the generation of electric power from biomass waste (Shimoyama *et al.*, 2008) and the treatment of wastewater (Liu *et al.*, 2004). Sediment-type MFCs have been examined for their ability to generate electricity in coastal sediment (CS) (Tender *et al.*, 2002) and rice paddy fields (Kaku *et al.*, 2008). In these MFCs, anodes are placed in sediment or soil, in which microbes oxidize organic matter and transfer electrons to the anodes (Kaku *et al.*, 2008; Kouzuma *et al.*, 2013). A recent study demonstrated that paddy-field MFCs exhibited the ability to generate as much as 140 mW m^–2^ of electric power (based on the projected area of the anode) (Ueoka *et al.*, 2016). CS-MFCs have been examined as *in situ* power sources for the operation of environmental sensors (Gong *et al.*, 2011) and the bioremediation of eutrophied sediment (Hamdan *et al.*, 2017).

Exoelectrogens are organisms that have the ability to transfer electrons extracellularly (Logan, 2009), and have been referred to as electrochemically active bacteria, anode-respiring bacteria, and electricigens in other studies (Lovley, 2006). Since exoelectrogens play a central role in MFCs, they have been extensively examined in order to obtain a clearer understanding of their ecology, physiology, and genetics. Numerous studies have performed the metabarcoding of 16S rRNA gene amplicons to characterize naturally occurring exoelectrogenic microbiomes (Zhi *et al.*, 2014). In addition, metagenomics/metatranscriptomics were conducted to identify and characterize uncultured exoelectrogens (Kouzuma *et al.*, 2018). A method for isolating exoelectrogens has also been developed (Ueoka *et al.*, 2018). Previous studies investigated model exoelectrogens, such as *Shewanella oneidensis* MR-1 (Kouzuma *et al.*, 2015) and *Geobacter sulfurreducens* PCA (Lovley, 2008), to elucidate the molecular mechanisms underlying extracellular electron transfer (EET). These organisms were shown to utilize multiheme *c*-type cytochromes as the major components in the EET pathways, while marked differences were also found in the components of their EET pathways (Ding *et al.*, 2016). Furthermore, recent studies clarified the regulatory mechanisms that control the expression of EET (Kasai *et al.*, 2015) and catabolic (Hirose *et al.*, 2018) pathways in *S. oneidensis*.

Previous studies identified bacteria affiliated with the genus *Desulfuromonas* as exoelectrogens that generate electricity in CS (Bond *et al.*, 2002; Holmes *et al.*, 2004), and *Desulfuromonas* bacteria were also shown to favor salt concentrations similar to those in seawater (Miyahara *et al.*, 2016). In these studies, acetate was used as the sole substrate for acclimatizing exoelectrogens, and, despite the practical importance of the remediation of eutrophied CS, limited information is currently available on the exoelectrogens involved in the decomposition of complex organic matter. According to previous findings obtained from paddy-field MFCs (Kouzuma *et al.*, 2013), it was hypothesized that the exoelectrogens that contribute to the conversion of complex organic matter into electricity in CS differ from those in acetate-fed MFCs. To examine this hypothesis in the present study, MFCs were operated using complex organic matter (starch, peptone, and fish extract) as substrates, and exoelectrogens that occurred from CS were identified.

## Materials and Methods

### CS samples

CS samples were obtained from four different sites in the Tokyo bay, Japan; these sites included the mouths of the Tamagawa river (at 35°32′ N, 139°45′ E) (TR), Obitsugawa river (at 35°24′ N, 139°54′ E) (OR), and Edogawa river (at 35°41′ N, 139°56′ E) (ER) as well as the Sanbanze tidal flat (at 35°40′ N, 139°57′ E) (ST). These CS samples were obtained in May 2017 and inoculated into MFCs immediately after sampling. Values for the temperature, oxidation/reduction potential (ORP), and pH at these CSs are summarized in supplemental material (Table S1).

### MFC set-up and operation

Single-chamber MFCs were used in the present study. Each MFC (approximately 15 mL in capacity) was equipped with an anode (5 cm^2^ in the projected area, made of graphite felt GF-80-3F; Sogo Carbon) and an air cathode (5 cm^2^ in the projected area). An air cathode was made according to a previously described method (Cheng *et al.*, 2006) and had four polytetrafluoroethylene layers on one side and a platinum catalyst layer (0.2 mg platinum cm^–2^, TEC10E20TPM; Tanaka Kikinzoku Kogyo) on the other side. Filter paper (No. 1004-240; GE Healthcare) was placed between the anode and cathode to prevent them from making contact.

An MFC was filled with 15 mL of an electrolyte containing (L^–1^) 29.5 g of soluble starch (FUJIFILM Wako Pure Chemical), 9.8 g of bactopeptone (Difco), 9.8 g of fish extract (Ehlrich; FUJIFILM Wako Pure Chemical), 30 g of NaCl, 3 mg of KH_2_PO_4_, 3 mg of CaCl_2_·2H_2_O, 4 mg of MgCl_2_·6H_2_O, 11 mg of NH_4_Cl, 50‍ ‍mg of NaHCO_3_, and 3 mL of a trace element solution used for DSMZ 663 medium (Deutsche Sammlung von Mikroorganismen und Zellkulturen); its pH was 7.0 and total chemical oxygen demand (COD_Cr_) concentration was approximately 5,000 mg L^–1^. The COD concentration of an electrolyte was adjusted by proportionally changing the concentrations of starch, bactopeptone, and fish extract. An MFC was inoculated with 0.3 g of CS, and its operation was initiated by connecting the anode and cathode via an external resister (*R*) at 30°C. Voltage (*E*) across the resistor was monitored using a data logger (GL800; Graphtec). Current was calculated from *E* and *R*, while current density (*J*) was based on the projected area of the anode (mA m^–2^). When voltage decreased, the electrolyte was replaced with fresh medium. The initial *R* was 5,000 Ω, and gradually decreased to 510 Ω with increases in current-generating capacities. MFC performances were evaluated by polarization analyses using a potentiostat (HSV-100; Hokuto Denko) as described previously (Logan *et al.*, 2006). The maximum power density (the peak in a power curve, *P*_max_) was assessed based on the projected area of the anode (mW m^–2^). The COD (COD_Cr_) concentration was measured using a COD reactor, COD 20–1,500 ppm range, and 250–15,000 kit (Hach).

### Metabarcoding of 16S rRNA gene amplicons

DNA was extracted from a piece of the anode, the electrolyte, and original CS using a FastDNA SPIN Kit for Soil (MP Bio). DNA quality was assessed by agarose gel electrophoresis and spectrophotometric analyses. Fragments of the V4 region in 16S rRNA genes were amplified from extracted DNA using universal primers (Caporaso *et al.*, 2010) according to protocols described elsewhere (Kouzuma *et al.*, 2017). In the forward primer, the rRNA gene sequence was connected to adaptor and tag sequences as described previously (Caporaso *et al.*, 2010). PCR products were purified using a QIAquick PCR purification kit (Qiagen), and after DNA concentrations had been assessed by measuring absorbance spectra, samples were mixed at the same concentration and subjected to pair-end sequencing with a MiSeq sequencer (Illumina) according to the protocol recommended by the manufacturer. Sequence reads greater than 230 bp were collected, and chimeric sequences were detected and removed using USEARCH via the uchime command (Edgar *et al.*, 2011). Sequences were clustered into operational taxonomic units with 97% similarity using QIIME (Caporaso *et al.*, 2010) and taxonomically classified by aligning these values with sequences in the Greengenes database (McDonald *et al.*, 2012).

### Isolation of exoelectrogens using electrode-plate culture (EPC) devices

Exoelectrogens were isolated from anode biofilms in MFCs using EPC devices as described previously (Ueoka *et al.*, 2018). The following procedures were conducted in an anaerobic chamber (BACTRON I; Sheldon). Agarose plates contained acetate (10‍ ‍mM) as a carbon source, while inorganic ingredients were the same as the electrolyte used in MFCs. Medium was also supplemented with Coomassie Brilliant Blue (10‍ ‍μg mL^–1^) to visualize small microbial colonies (Ueoka *et al.*, 2018). A biofilm suspension was streaked on an agarose plate and covered with a transparent working electrode (WE) made of fluorine-doped tin oxide. The anode potential was poised at –0.2 V vs. an Ag/AgCl reference electrode (HX-R5; Hokuto Denko) using a potentiostat (VMP3; Bio-Logic), and colony formation and current generation were observed.

A colony that formed in the EPC device was picked up using a needle and inoculated into a single-chamber electrochemical cell (EC, capacity of 15 mL) equipped with a graphite-felt WE (1.5×1.5 cm, Sogo Carbon), platinum-wire counter electrode (12‍ ‍cm), and Ag/AgCl reference electrode. EC contained 15 mL of the electrolyte used for the above-described MFCs, while acetate (10‍ ‍mM) was the sole carbon source and electron donor. When current was generated, the electrolyte was sampled and streaked onto agarose plates containing the electrolyte supplemented with acetate (10‍ ‍mM) and ferric citrate (20‍ ‍mM) to check the purity of cultures (Ueoka *et al.*, 2018). Purity was confirmed by checking the colony morphology and sequence purity of PCR-amplified 16S rRNA gene fragments.

### Characterization of isolates

Colonies picked from agarose plates were grown in liquid media, and the resultant cells were used for the sequencing of partial 16S rRNA genes as described previously (Ueoka *et al.*, 2018). The sequences elucidated for isolates were analyzed using the Blast program (NCBI, http://www.ncbi.nlm.nih.gov/), and a phylogenetic tree was constructed using MEGA5 software (Tamura *et al.*, 2011).

ECs were used to measure the exoelectrogenic activity of an EPC isolate with different substrates. An EC was filled with the electrolyte supplemented with a carbon source (glucose, acetate, propionate, or lactate) at 10‍ ‍mM, and, after autoclaving and nitrogen purging, it was inoculated with an isolated strain. The electrodes were connected to the potentiostat, and WE was poised at +0.3‍ ‍V vs. the Ag/AgCl reference electrode to examine current generation. Current density (*J*) was calculated based on the projected area of WE (μA cm^–2^), while *J*_max_ (μA cm^–2^) was the maximum value of *J* during the measurement.

### Sequence deposition

The raw sequences generated in the metabarcoding analysis have been deposited in the DDBJ Sequence Read Archive database under accession number DRA009342. 16S rRNA gene sequences abundantly detected by metabarcoding are deposited in the DDBJ, EMBL, and NCBI nucleotide sequence databases under accession numbers LC517400 to LC517452. The 16S rRNA gene sequence for strain OR-1 is deposited under accession number LC512020.

## Results and Discussion

### Electricity generation in CS-MFCs

**Fig. 1.**
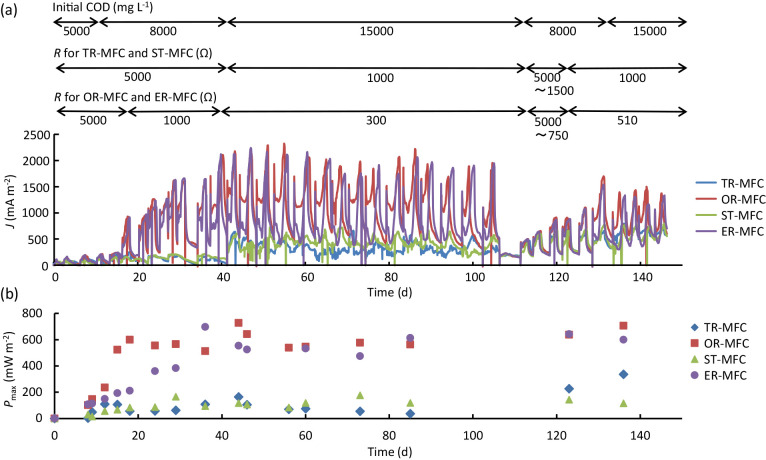
Electricity generation in CS-MFCs. (a) Changes in *J* (a) and *P*_max_ (b). COD concentrations in refreshed media and external resisters for CS-MFCs are indicated above the *J* curves. On day 106, portions of anodes were cut from these MFCs, and they were re-started.

The operation of MFC inoculated with either of the four different CSs, namely, TR-MFC, OR-MFC, ER-MFC, and ST-MFC (collectively, CS-MFCs), was initiated in the presence of the electrolyte containing the complex substrates at a COD concentration of approx. 5,000 mg L^–1^. When the cell voltage decreased, the electrolyte was replaced with fresh medium. COD concentrations were then increased as shown in Fig. 1a. The initial external resistance was 5,000‍ ‍Ω, and this subsequently decreased with increases in cell voltage (Fig. 1a). Although these CS-MFCs were operated similarly, current generation from two MFCs (OR-MFC and ER-MFC) was superior to that from the other two (TR-MFC and ST-MFC). As shown in Table S1, CSs from OR and ER exhibited lower ORP values than those for CSs from TR and ST, suggesting that ORP is an important factor for assessing the suitability of CSs for electricity generation in MFCs.

CS-MFCs were routinely subjected to polarization analyses to obtain *P*_max_ values when cell voltages were sufficiently high (Fig. 1b). *P*_max_ values gradually increased for approximately one month after the initiation of operation, and subsequently became relatively stable. After becoming stable, two MFCs (OR-MFC and ER-MFC) exhibited relatively high *P*_max_ values (approximately 600 mW m^–2^), while *P*_max_ values for the other two MFCs (TR-MFC and ST-MFC) were low (approximately 100 mW m^–2^). Therefore, further experiments were needed to clarify how the microbiomes established in these CS-MFCs were different and correlated with power outputs.

### Microbiomes in CS-MFCs

**Fig. 2.**
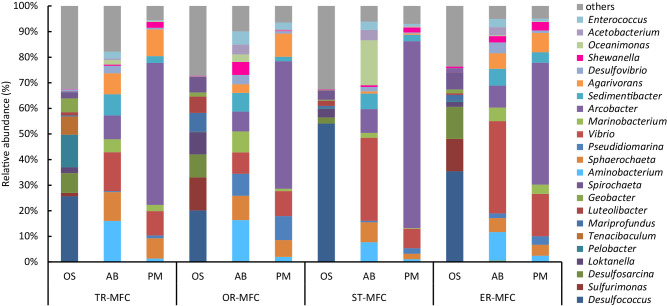
Metabarcoding of 16S rRNA gene amplicons for bacterial populations in original sediments (OS), anode biofilms (AB), and planktonic microbes (PM) in CS-MFCs. Sequences were classified at the genus level.

Microbiomes in the original marine sediments (OS), anode biofilms (AB), and planktonic microbes (PM) in CS-MFCs on day 106 (see Fig. 1a) were subjected to the metabarcoding of 16S rRNA gene amplicons in order to comparatively characterize community members (Fig. 2). Bacteria present in MFCs were completely different from those in OS, among which those related to *Arcobacter* were particularly abundant in PM in all CS-MFCs. The genus *Arcobacter* is known to include pathogens that have been detected and isolated from coastal environments (Rathlavath *et al.*, 2017), while another study isolated electrochemically active *Arcobacter* strains from MFCs (Fedorovich *et al.*, 2009).

Some bacteria specifically increased in the AB fractions and included *Aminobacterium*, *Sedimentibacter*, and *Vibrio* (these were >1% of the total sequences). Previous studies suggested that bacteria occurring specifically in AB are generally responsible for current generation in MFCs (Kouzuma *et al.*, 2018). The relative abundance of these bacteria, in addition to known exoelectrogens, including *Shewanella*, *Geobacter*, and *Desulfuromonas*, in each library are summarized in supplemental material (Table S2). As shown in this table, *Geobacter* and *Desulfuromonas* were minor in these CS-MFCs.

*Aminobacterium* is a genus of bacteria in the family *Synergistaceae* and known to be strict anaerobes that ferment amino acids to produce acetate (Baena *et al.*, 1998). These bacteria have been detected in anaerobic digesters, *e.g.*, solid-state biogas reactors, and may preferentially grow in association with solid materials (Li *et al.*, 2013). In the present study, *Aminobacterium* were detected in close association with AB, while this genus weakly correlated with power outputs from MFCs. Relatively diverse sequences were detected for *Aminobacterium*, and the phylogenetic relationships between these sequences and those stored in the databases are shown in supplemental material (Fig. S1). *Sedimentibacter* are affiliated with the phylum *Firmicutes* and are strict anaerobes that ferment amino acids and pyruvate to produce acetate and propionate (Breitenstein *et al.*, 2002). These bacteria may occur in MFCs by consuming amino acids derived from bactopeptone and fish extract as carbon and energy sources. On the other hand, *Vibrio* is a genus in the class *Gammaproteobacteria* (Farmer *et al.*, 2015), and a previous study identified a *Vibrio* strain in MFC that exhibited exoelectrogenic activity (Li *et al.*, 2019).

*Shewanella* was detected with abundance in CS-MFCs (Fig. 2 and Table S2). These bacteria are affiliated with the class *Gammaproteobacteria* and known to be facultative anaerobes with the ability to metabolize a number of inorganic and organic compounds (Venkateswaran *et al.*, 1999). Bacterial strains affiliated with the genus *Shewanella* are also known to be exoelectrogens that grow by generating electricity in MFCs (Kouzuma *et al.*, 2015). A previous study reported that in MFCs treating high molecular-weight organics, fermentative bacteria outgrew exoelectrogenic bacteria, and, in many cases, exoelectrogenic bacteria did not exceed 10% of the total population (Miyahara *et al.*, 2013). In the present study, since *Shewanella* was more abundantly detected in CS-MFCs generating high power densities (OR-MFC and ER-MFC) than in low-power MFCs (TR-MFC and ST-MFC) (supplemental material Fig. S2), it is reasonable to deduce that these bacteria contributed to electricity generation in CS-MFCs. On the other hand, although *Vibrio* was more abundantly detected in AB than *Shewanella*, they were abundant in all CS-MFCs irrespective of power outputs (Table S2). Although multiple species of exoelectrogens are involved in electricity generation in CS-MFCs treating complex organic matter, the present results suggest that *Shewanella* is one of these exoelectrogens contributing CS-MFCs.

### Isolation and characterization of exoelectrogens

To further characterize exoelectrogens in CS-MFCs, we attempted to isolate exoelectrogens from CS-MFCs with a particular focus on *Shewanella* strains detected in high-power MFCs (Fig. 2). Suspensions of AB obtained from OR-MFC were subjected to EPC devices (Ueoka *et al.*, 2018), and ECs were inoculated with the colonies that formed in EPCs to confirm whether they were able to generate electric currents. Microbes were subsequently isolated from current-generating ECs using agar plates containing ferric citrate as the electron acceptor. The results obtained showed that we successfully isolated a *Shewanella* strain from OR-MFC, which we named strain OR-1.

**Fig. 3.**
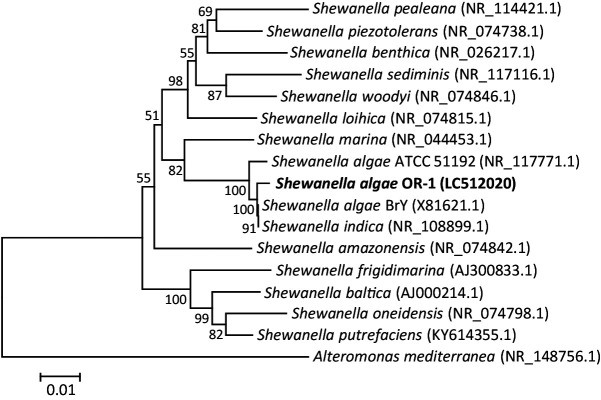
A phylogenetic tree based on 16S rRNA gene sequences showing relationships among bacterial strains in the genus *Shewanella*. *Alteromonas mediterranea* is used as the outgroup. Bootstrap values (100 trials) are indicated at branching points. Sequence divergence is indicated with bars, and accession numbers are shown in parentheses.

Analyses of 16S rRNA genes indicated that the sequence of OR-1 was identical to the major sequence type of *Shewanella* detected in AB in the metabarcoding analyses. Phylogenetic analyses showed that OR-1 was affiliated with *S. algae* and closely related to *S. algae* strain BrY (99% identical nucleotide sequence) (Fig. 3). This bacterium was isolated from estuary sediment (Caccavo *et al.*, 1992). BrY excretes melanin, which serves as a soluble electron shuttle for Fe(III) oxide reduction (Turick *et al.*, 2002), while the capacity of *S. algae* strains to generate electricity in MFC has not yet been examined. OR-1 is also closely related to *S. indica* (Verma *et al.*, 2011), and a recent phylogenomic study suggested that *S. indica* needs to be included in *S. algae* (Thorell *et al.*, 2019).

**Fig. 4.**
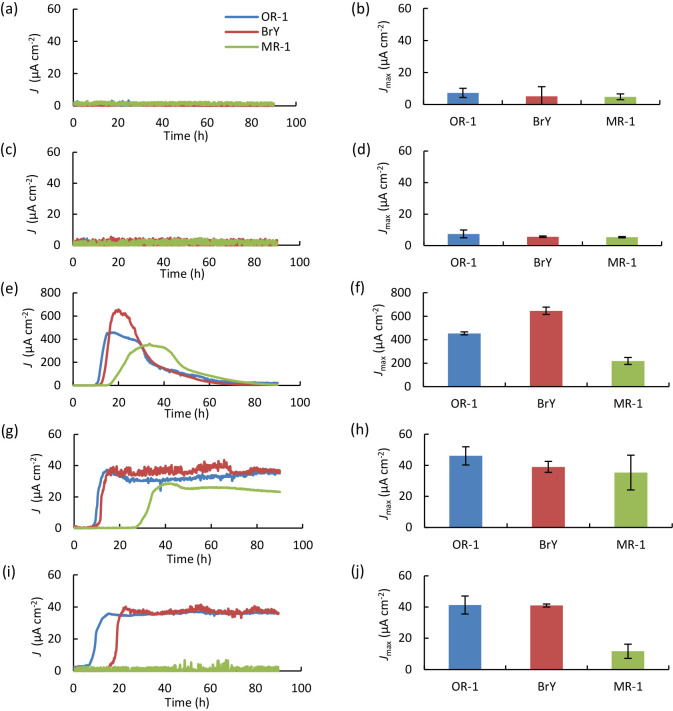
Current generation by *Shewanella* sp. OR-1, *Shewanella algae* BrY, and *Shewanella oneidensis* MR-1. Representative *J* curves (a, c, e, g, and i) and mean *J*_max_ values (*n*=3, bars indicate SDs) (b, d, f, h, and j) are shown. The substrates used (10‍ ‍mM) were no substrate (a, b), glucose (c, d), lactate (e, f), propionate (g, h), and acetate (i, j).

We examined current generation by strain OR-1 with glucose, lactate, propionate, and acetate as substrates (Fig. 4); in these analyses, we also examined *S. algae* BrY and *S. oneidensis* MR-1 for comparison. Strain MR-1 is a representative of *Shewanella* exoelectrogens, the current-generating mechanisms of which have been extensively examined (Kouzuma *et al.*, 2015). Fig. 4 shows that the three strains were unable to generate current from glucose. Lactate was the best substrate among those examined, while *S. algae* strains generated higher *J*_max_ than that of MR-1. While the three *Shewanella* strains exhibited the ability to generate current from propionate, the *S. algae* strains, but not MR-1, generated current from acetate.

The generation of current from acetate by *Shewanella* strains was unexpected because *Shewanella* strains perform the incomplete oxidation of organic matter to produce acetate as the end product under anaerobic conditions, including electricity-generating conditions (Lanthier *et al.*, 2008). Therefore, catabolic pathways in *S. algae* strains differ from those in authentic *Shewanella* strains, including strain MR-1, and it will be interesting to investigate their catabolic pathways. A recent study demonstrated that a novel isolate of *S. algae* exhibited the ability to oxidize acetate with manganese(III) as the sole electron acceptor (Szeinbaum *et al.*, 2017). Therefore, acetate oxidation under anaerobic conditions may be a common feature of *S. algae* strains that is distinct from other *Shewanella* strains.

Metabarcoding analyses (Fig. 2) showed that some fermentative bacteria that produce acetate and propionate as fermentation products occurred in CS-MFCs. Therefore, we examined current generation from acetate and propionate and found that strain OR-1 exhibited the ability to generate electricity from these compounds (Fig. 4). Based on these results, in CS-MFCs treating complex organic matter, exoelectrogens, such as *Shewanella* sp. OR-1, generate electricity by using fermentation products, such as acetate and propionate, excreted by fermentative bacteria, including *Aminobacterium* and *Sedimentibacter*, while other bacteria may decompose complex organic matter into monomers.

## Conclusions

Although previous studies identified *Desulfuromonas* bacteria as marine exoelectrogens (Bond *et al.*, 2002; Holmes *et al.*, 2004), the present study demonstrated that these bacteria did not occur in CS-MFCs treating complex organic matter. The results obtained herein showed that other bacteria occur in CS-MFCs and contribute to electricity generation. These bacteria include *S. algae* relatives with the ability to generate electricity from some organic acids, including acetate. These exoelectrogens may constitute syntrophic interactions with fermentative bacteria for the conversion of complex organic matter into electricity. Further studies are needed to detect and isolate diverse coastal and marine exoelectrogens that will grow under different MFC operational conditions.

## Citation

Inohana, Y., Katsuya, S., Koga, R., Kouzuma, A., and Watanabe, K. (2020) *Shewanella algae* Relatives Capable of Generating Electricity from Acetate Contribute to Coastal-Sediment Microbial Fuel Cells Treating Complex Organic Matter. *Microbes Environ ***35**: ME19161.

https://doi.org/10.1264/jsme2.ME19161

## Supplementary Material

Supplementary Material
