# Supplementary material for: Shewanella algae Relatives Capable of Generating Electricity from Acetate Contribute to Coastal-Sediment Microbial Fuel Cells Treating Complex Organic Matter
Source: Microbes Environ. 2020 Mar 6;35(2):ME19161. doi: 10.1264/jsme2.ME19161 (PMC7308575; doi:10.1264/jsme2.ME19161)
Supplement: Supplementary file 1 — Supplementary Material [file 35_19161_s1.pdf]

Supplemental material

Table S1. Environmental parameters (temperature, ORP and pH) for CSs used as inocula for MFCs.

Parameter	TR	OR	ST	ER
Temperature (℃)	25	25	25	19
ORP (mV)	-45	-260	10	-183
pH	8.1	8.1	8.8	8.0

Table S2. Number of sequences detected by the metabarcoding showing relative abundances of sequences affiliated with the genera *Shewanella*, *Geobacter*, *Desulfuromonas*, *Aminobacterium*, *Sedimentibacter*, *Arcobacter* and *Vibrio* in each library.

(a) TR-MFC

	OS	AB	PM
Shewanella	8	119	729
Geobacter	346	7	1
Desulfuromonas	1	-	-
Aminobacterium	1	4076	407
Sedimentibacter	-	2112	850
Arcobacter	18	2382	17531
Vibrio	5	3880	3005

(b) OR-MFC

	OS	AB	PM
Shewanella	19	893	79
Geobacter	233	-	-
Desulfuromonas	10	-	-
Aminobacterium	1	2814	680
Sedimentibacter	-	1284	607
Arcobacter	22	1363	17392
Vibrio	2	1459	3413

(c) ST-MFC

ST-MFC	OS	AB	PM
Shewanella	20	212	855
Geobacter	48	4	-
Desulfuromonas	1	1	-
Aminobacterium	-	2538	459
Sedimentibacter	-	1983	1092
Arcobacter	9	3088	32125
Vibrio	7	10692	3346

(d) ER-MFC

ER-MFC	OS	AB	PM
Shewanella	83	583	1393
Geobacter	201	130	1
Desulfuromonas	-	3	-
Aminobacterium	-	2629	988
Sedimentibacter	-	1562	1699
Arcobacter	210	2023	19662
Vibrio	9	8519	6815

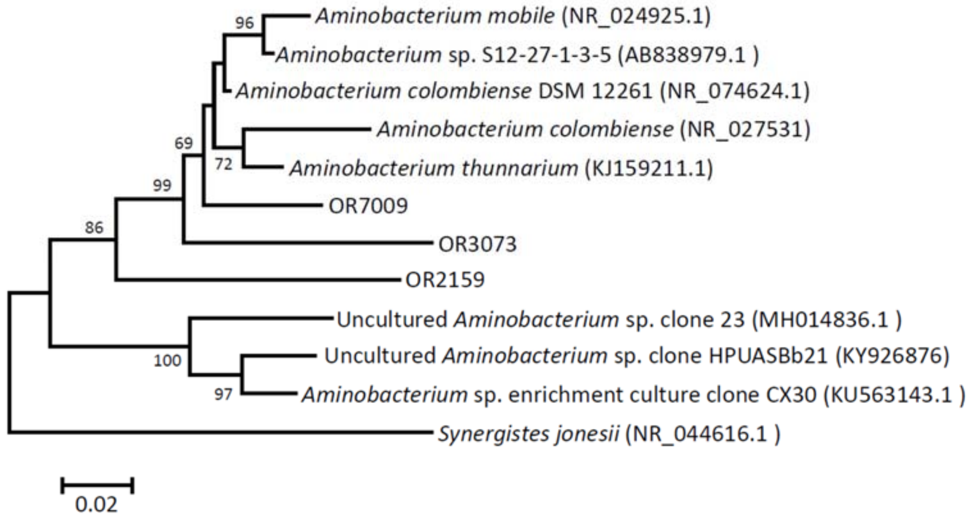

Fig. S1. A phylogenetic tree showing relationships between *Aminobacterium* sequences abundantly detected in the present study (OR7009, OR3073 and OR2159) and those stored in the databases. Accession numbers are shown in parentheses. Numbers at branch nodes are bootstrap values (only >50 are shown). A bar represents substitution per site.

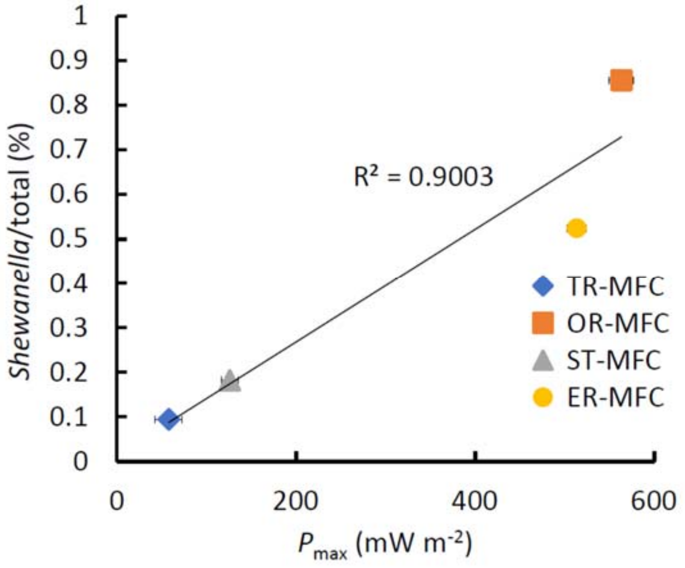

Fig. S2. Relationship between P_{max} of CE-MFCs and relative abundance of *Shewanella* sequences in AB fractions. Error bars represent SDs.
